# ﻿A taxonomic study of *Psyllaephagus* Ashmead (Hymenoptera, Encyrtidae) from China

**DOI:** 10.3897/zookeys.1184.109476

**Published:** 2023-11-23

**Authors:** Boyu Zou, Hongying Hu, Lanwei Zhang, Yanzhou Zhang

**Affiliations:** 1 College of Life Science and Technology, Xinjiang University, Urumqi, Xinjiang 830017, China Xinjiang University Urumqi China; 2 Institute of Zoology, Chinese Academy of Sciences, Beijing 100101, China Institute of Zoology, Chinese Academy of Sciences Beijing China

**Keywords:** Aphalaridae, Carsidaridae, Homotomidae, Psyllidae, Triozidae

## Abstract

Fifteen species of *Psyllaephagus* from China are studied. Three species, *P.clavus* Zou & Zhang, **sp. nov.**, *P.obliquus* Zou & Zhang, **sp. nov.**, and *P.tangae* Zou & Zhang, **sp. nov.**, are described as new to science. A diagnosis or a description/redescription, figures of the characters, as well as the known distribution and hosts of each species are provided. A dichotomous key is also given to facilitate the identification of species.

## ﻿Introduction

In the family Encyrtidae, *Psyllaephagus* Ashmead, 1900 is one of the largest genera with approximately 246 recognized species (Noyes 2019). Most species of *Psyllaephagus* are primary parasitoids of psylloids (Hemiptera: Psylloidea), whilst some are secondary (Noyes and Hanson 1996; Trjapitzin 2012). Among *Psyllaephagus*, three species from Australia have been successfully used for biological control of psylloids. *Psyllaephaguspilosus* Noyes, 1988 from Australia was introduced and released in California and European countries to control *Ctenarytainaeucalypti* Maskell, 1890 (Dahlsten et al. 1998; Hodkinson 1999; Chauzat et al. 2002). *Psyllaephagusbliteus* Riek, 1962 was introduced and released in California to control *Glycaspisbrimblecombei* Moore, 1964 (Daane et al. 2005). *Psyllaephagusyaseeni* Noyes, 1990 was introduced into Hawaii and south-east Asia for control of *Heteropsyllacubana* Crawford, 1914 (Beardsley and Uchida 1990).

The greatest number of species occur in Australia where 100 species of *Psyllaephagus* have been described, although there may be as many as 1,000 species (Noyes and Hayat 1984). Species of *Psyllaephagus* in the Costa Rican region were studied by Noyes and Hanson (1996). Girault (1915) and Riek (1962) reported a number of species from Australia. Trjapitzin (1989) revised *Psyllaephagus* and provided a key to 57 species distributed in Palaearctic region. Twenty species of *Psyllaephagus* are recorded from India (Singh 1996; Hayat 2003), and 22 species from Africa (Prinsloo 1981). The Chinese fauna have been studied by Tan and Zhao (1999), Xu et al. (2000a, b), Tang et al. (2016), and Wu et al. (2021). Several species names used in a Master’s thesis by Li (2010) and a PhD thesis by Zhang (2001) are unavailable because these theses have not been formally published.

The present paper is intended as a comprehensive taxonomic study of all known species from China. However, we were not able to examine the specimens of *Psyllaephagusbelanensis* (Hoffer, 1963) mentioned by Ma (2004) and *P.belanensis* is therefore not included in this study.

## ﻿Materials and methods

Many of the specimens included the present study were reared from psylloids collected from different regions of China. Twigs and/or leaves with psylloids were collected and brought back to the Key Laboratory of Zoological Systematics and Evolution (Institute of Zoology, Chinese Academy of Sciences, **IZCAS**) and college of Life Science and Technology, Xinjiang University (**ICXU**). Twigs and/or leaves with psylloids nymphs were kept in nylon bags (100 mesh) for at least one month to allow parasitoids to emerge. Parasitoids were collected and preserved in ethanol (99%). The remaining specimens were collected by using a sweeping net (see Noyes 1982). Part of the newly collected specimens were card-mounted, and others had been dissected and mounted on slides generally following the method described by Noyes (1982). Observation and measurement of specimens were made with a Nikon SMZ-168 stereomicroscope. Microphotographs were taken with a Canon EOS550D digital camera connected to a Leica DM-2500 microscope, and photographs of card mounted specimens were produced using a Nikon D7000 digital camera coupled to a Nikon SMZ-1500 stereomicroscope. All materials examined are deposited in IZCAS and ICXU.

Morphological terminology and abbreviations follow that of Noyes and Hanson (1996) and Gibson (1997) with some modifications. Absolute measurements were used for body length. Abbreviations used in the text are as follows: **HW**, maximum head width; **HL**, minimum head length; **FV**, the width of the frontovertex; **OOL**, the minimum distance between the eye margin and the nearest posterior ocellus; **POL**, the minimum distance between the posterior ocelli; **SL**, the length of the scape; **SW**, maximum width of the scape; **F1** through **F6**, 1^st^ to 6^th^ funicular segments; **MSL**, mesoscutum length; **MSW**, mesoscutum width; **MT**, mid tibia length; **MV**, marginal vein length; **PMV**, postmarginal vein length; **STV**, stigmal vein length; **FVL**, the maximum length of fore wing; **FVW**, the maximum width of fore wing; **OL**, length of ovipositor; **GL**, length of third valvula.

Abbreviations of depositories: **IZCAS**, Institute of Zoology, Chinese Academy of Sciences, Beijing, China; **CNEP**, Collection of Natural Enemies of Pests, Hubei University, Hubei, China; **ICXU**, Collection of Life Science and Technology, Xinjiang University, Xinjiang, China; **TARI**, Insect Museum, Taiwan Agricultural Research Institute, Taiwan, China; **ZUIE**, Institute of Applied Entomology, Zhejiang University, Zhejiang, China; **TJAU**, Insect Collection of Tianjin Agricultural University, Tianjin, China; **ZISP**, Zoological Institute, St Petersburg, Russia.

## ﻿Results

### ﻿Genus Psyllaephagus

Taxon classificationAnimaliaHymenopteraEncyrtidae

Ashmead, 1900

84870FE0-6D49-565B-AB5D-DC53029C41C4

Psyllaephagus Ashmead, 1900: 382. Type species: Encyrtuspachypsyllae Howard, 1885 by original designation. Type locality U.S.A.
Mirocerus Ashmead, 1904: 309. Type species: Miroceruspeyelae Ashmead, by original designation. Type locality Sri Lanka. Synonymized with Psyllaephagus by Trjapitzin (1978: 636).
Calocerineloides Girault, 1913: 11. Type species: Calocerineloidesramosa Girault, by original designation. Type locality Australia. Synonymized with Psyllaephagus by Noyes and Hayat (1984: 330).
Epanagyrus Girault, 1915: 160. Type species: Epanagyruspunctatiscutum Girault, by original designation. Type locality Australia. Synonymized with Psyllaephagus by Noyes and Hayat (1984: 330).
Neanagyrus Girault, 1915: 174. Type species: Neanagyruscapitatus Girault, by original designation. Type locality Australia. Synonymized with Psyllaephagus by Dahms and Gordh (1997: 305).
Anagyropsis Girault, 1917: 136. Type species: Anagyropsispurpureus Girault, by original designation. Type locality Australia. Synonymized with Psyllaephagus by Noyes and Hayat (1984: 330).
Metaprionomitus Mercet, 1921: 260. Type species: Metaprionomitusintermedius Mercet, by original designation. Type locality Spain. Synonymized with Psyllaephagus by Trjapitzin (1967: 192).
Shakespearia Girault, 1928: 3. Type species: Shakespeariaflabellata Girault, by monotypy. Type locality Australia. Synonymized with Psyllaephagus by Noyes and Hayat (1984: 330).
Psyllencyrtus Tachikawa, 1955: 63. Type species: Psyllencyrtussyntomozae Tachikawa, by original designation. Type locality Japan. Synonymized with Psyllaephagus by Tachikawa (1981: 88).
Calluniphilus Erdös, 1961: 413. Type species: Calluniphilusvendicus Erdös, by monotypy. Type locality Australia. Synonymized with Psyllaephagus by Graham (1969: 248–249).
Ooencyrtoides Hoffer, 1963: 235. Type species: Ooencyrtoidesalbopilosus Hoffer, by original designation. Type locality Czech. Synonymized with Psyllaephagus by Graham (1969: 248–249).
Propsyllaephagus Blanchard in De Santis, 1964: 235. Type species: Propsyllaephagustrellesi Blanchard, by monotypy. Type locality Argentina. Synonymized with Psyllaephagus by Noyes (1979: 165).
Mercetia Bakkendorf, 1965: 139. Type species: Copidosomalusitanicum Mercet, 1921 by original designation. Type locality Spain. Synonymized with Psyllaephagus by Graham (1969: 248–249).
Kaszabicyrtus Szelényi, 1971: 389. Type species: Kaszabicyrtusacutigastris Szelényi, by original designation. Type locality Mongolia. Synonymized with Psyllaephagus by Trjapitzin and Gordh (1978: 636).

#### Other citations.

Trjapitzin 1978, 1989; Noyes and Hanson 1996; Myartseva 1980, 1984; Zhang and Huang 2004; Japoshvili et al. 2016.

#### Diagnosis.

**Figure 1.**
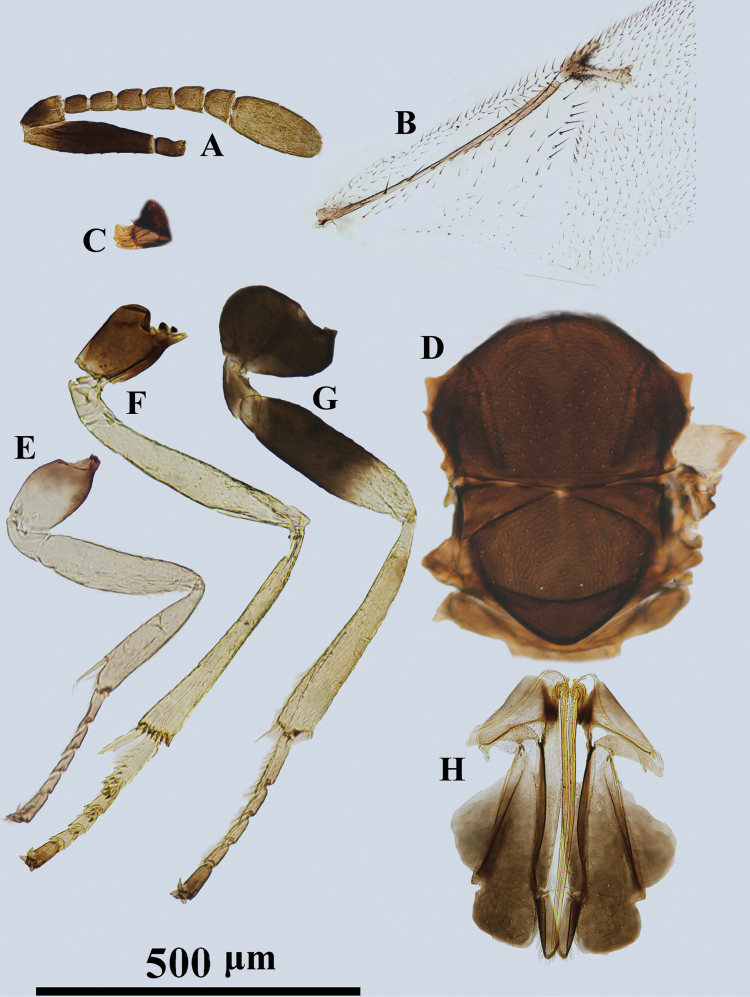
*Psyllaephagusarenarius* ♀ **A** antenna **B** mandible **C** fore wing **D** mesonotum **E** fore leg **F** mid leg **G** hind leg **H** ovipositor.

**Figure 2.**
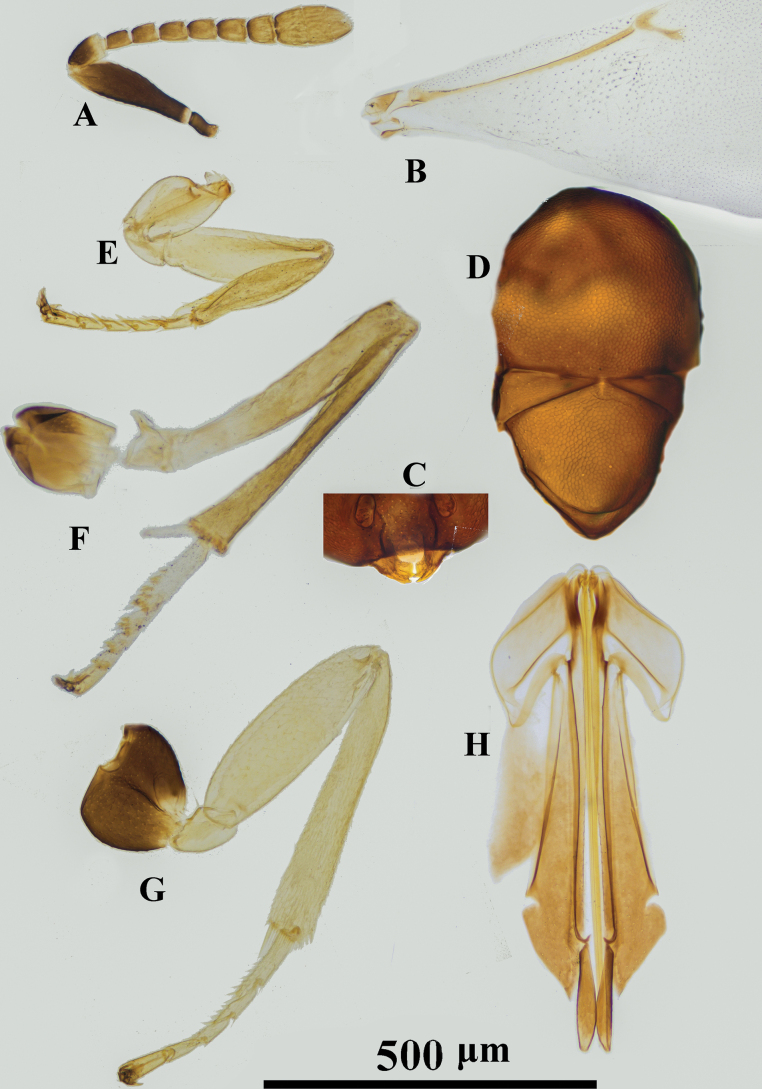
*Psyllaephaguscaillardiae* ♀ **A** antenna **B** mandible **C** fore wing **D** mesonotum **E** fore leg **F** mid leg **G** hind leg **H** ovipositor.

Female. Body length 0.8–3.0 mm usually dark brown with green or blue sheen and with metallic luster; occiput margin often rounded, rarely carinate; mandible with one tooth and a truncation (Fig. 1B), or with two teeth and a truncation (Fig. 2B), rarely with three teeth; funicle 6-segmented; clava often 3-segmented or entire, rarely 2-segmented; fore wing fully developed, often hyaline, rarely with a smoky spot under marginal vein and stigmal vein; marginal vein usually punctiform or slightly longer than wide (Fig. 15C), rarely 2–3× as long as broad; postmarginal vein usually shorter than stigmal vein, occasionally absent, in some species as long as or even longer than stigmal vein; mesopleuron in side view clearly separated from base of metasoma by propodeum; mid tibia spur usually shorter than basal tarsus; hypopygium very rarely reaching apex of metasoma; ovipositor hidden, slightly to strongly exserted. Male essentially similar in appearance except for antennae and genitalia: the funicle varying from whiplike with long setae to flattened with short setae, clava entirely; the genitalia usually developed and exserted.

### ﻿ Psyllaephagus arenarius

Taxon classificationAnimaliaHymenopteraEncyrtidae

Trjapitzin, 1967

AD8FFCEB-7E58-55B2-A877-FC81CC0E4571

Fig. 1

Psyllaephagus arenarius Trjapitzin, 1967: 192. Holotype ♀, Russia (ZISP), not examined.
Psyllaephagus arenarius Trjapitzin, 1968: 96–97, 1978: 301, 1981: 14–16; 1989: 256; Zhang et al. 2017: 842–846.

#### Material examined.

China – **Ningxia** • 13♀♀, 12♂♂, Zhongwei, 09.Jul.2016, leg. PX Wu, ex. *Bactericeragobica* on *Lyciumbarbarum*; Zhonwei, 17.Aug.2016, PX Wu leg., ex. *Bactericeragobica* on *Lyciumbarbarum*; 22♀♀, 37♂♂, 11♀♀, Jixian, 17.Apr.2017, leg. X Zhang; **Shandong** • 10♀♀, 3♂♂, Junan, 11.Jun.2018, leg. YZ Zhang.

#### Diagnosis.

Female. Body length ~ 1.3 mm; mid and hind coxae dark brown, basal ~ 2/3 of hind femur dark brown; F1–F5 usually a little longer than wide, F6 quadrate (Fig. 1A); ovipositor not or hardly exserted (Fig. 1H), OL ~ 1.1× as long as MT.

#### Description.

**Female. *Body*** length 1.2–1.6 mm. Frontovertex and scrobe with blue-violet sheen; mesoscutum, axillae metallic green; scutellum with a golden-green sheen; metasoma with metallic green sheen; scape and pedicel dark brown, but apex of 1/5 scape and 1/3 pedicel paler; funicle and clava yellowish brown (Fig. 1A); tegula pale yellow, except apex dark brown; legs yellow, except basal 1/4 of fore coxa brown, mid and hind coxae dark brown, basal 2/3 of hind femur dark brown (Fig. 1E–G); wings hyaline, venation brown (Fig. 1B).

***Head*** with reticulate sculpture; HW 2.1× HL in dorsal view, and ~ 3.0× FV; ocelli form acute angle of slightly < 90°; OCL ~ 1.7× diameter of posterior ocellus; OOL ~ 1.0× diameter of posterior ocellus; POL ~ 2.7× diameter of posterior ocellus; mandible with one tooth and a broad truncation (Fig. 1C); upper margin of torulus below level of lower eye margin; antenna with scape ~ 4× as long as wide; pedicel ~ 1.9× as long as wide; F1–F5 longer than broad, F6 subquadrate; clava 3-segmented, as long as F4–F6 combined, nearly 2.2× as long as wide with apex rounded. Relative measurements: HW 22; HL 10.4; FV 7.3; OOL 2; POL 5.4; OCL 3.4; SW 2.5; SL 10.5.

***Mesoscutum*** slightly convex; mesoscutum and scutellum with similar reticulate sculpture to that on frontovertex (Fig. 1D); pronotum ~ 0.25× as long as wide; MSW ~ 1.6× MSL; scutellum ~ 1.5× as long as wide; fore wing ~ 2.3× as long as wide; marginal vein punctiform; stigmal vein ~ 5.5× as long as wide, STV ~ 2.5× PMV; basal cell almost bare; mid tibial spur ~ 0.7× as long as basitarsus. Relative measurements: FWL 35.5; FWW 19.7.

***Metasoma*** ~ 0.8× as long as mesosoma; cercal plates located in the 1/2 basal of metasoma; hypopygium reaching 2/3 of metasoma; ovipositor not exserted (Fig. 1H), OL ~ 5.0× GL, and 1.1× MT.

**Male.** Length 1.0–1.3 mm; generally similar to female except antenna and genitalia. All funicle segments longer than broad and clothed with long setae, usually longer than funicle segments

#### Hosts.

*Acaerusturkestanicus* Low, 1881 (Hemiptera: Psyllidae), *Bactericeragobica* Loginova, 1972 (Hemiptera: Triozidae).

#### Distribution.

China (Ningxia, Shandong, Tianjin); other countries: Russia (Tajikistan).

#### Comments.

The specimens studied here agree well with the original description of *P.arenarius*, except the length of clava is slightly shorter than F3–F6 combined; in the original description, the clava is approximately as long as F3–F6 combined.

### ﻿ Psyllaephagus caillardiae

Taxon classificationAnimaliaHymenopteraEncyrtidae

Sugonjaev, 1968

2E819A4A-2403-5C83-B862-9E6536326A1B

Fig. 2

Psyllaephagus caillardiae Sugonjaev, 1968: 592. Holotype ♀, Russia (ZISP), not examined.
Psyllaephagus caillardiae : Danzig and Sugonjaev 1969: 116–124; Myartseva 1984: 145, 233; Tang et al. 2016: 65.

#### Material examined.

China– **Xinjiang** • 15♀♀, 11♂♂, Shihezi, 21.Jul.2012, ex. *Caillardiaazurea* on *Haloxylonpersicum*, leg. HY Hu; 19♀♀, 7♂♂, Karamay, 25.Jul.2016, ex. *Caillardianotata* on *Haloxylonammodendron*, leg. HY Hu’s group; 17♀♀, 3♂♂, 18.Aug.2018, Fukang, leg. HY Hu’s group.

#### Diagnosis.

Female. Body length ~ 1.5 mm; legs yellow, but basal ~ 1/2 of mid coxae and entire hind coxae dark brown (Fig. 2E–G); ocelli forming an angle of ~ 90°; mesoscutum and scutellum with similar reticular sculpture (Fig. 2D); ovipositor distinctly exserted, the exserted part ~ 1/4 of metasoma; OL ~ 1.6× MT (Fig. 2H).

#### Description.

See Tang et al. (2016).

#### Hosts.

*Caillardiaazurea* Loginova, 1956 and *Caillardiarobusta* Loginova, 1956 (Hemiptera: Aphalaridae).

#### Distribution.

China (Xinjiang); other countries: Kazakhstan, Mongolia, Tajikistan, Turkmenistan, Uzbekistan.

#### Comments.

The specimens examined here agree well with the original description of *P.caillardiae*, except having the basal ~ 1/2 of mid coxa dark brown; in the original description, the mid coxae were described as yellow.

### ﻿ Psyllaephagus clavus

Taxon classificationAnimaliaHymenopteraEncyrtidae

Zou & Zhang sp. nov.

3AC9F5E0-E99E-52EA-9AA2-7757829612A7

https://zoobank.org/DC2C6224-5426-4FCF-8620-46CFC916A97C

Fig. 3

Psyllaeohagus nartshukae Trjapitzin: Tang et al. 2016: 72–73 (misidentification).

#### Type material.

***Holotype*** ♀ [on slide], China –**Xinjiang**, Shihezi, 21.Aug.2012, by sweeping, leg. HY Hu group (deposited in ICXU). ***Paratypes*** 4♀♀, same data as holotype.

#### Diagnosis.

**Figure 3.**
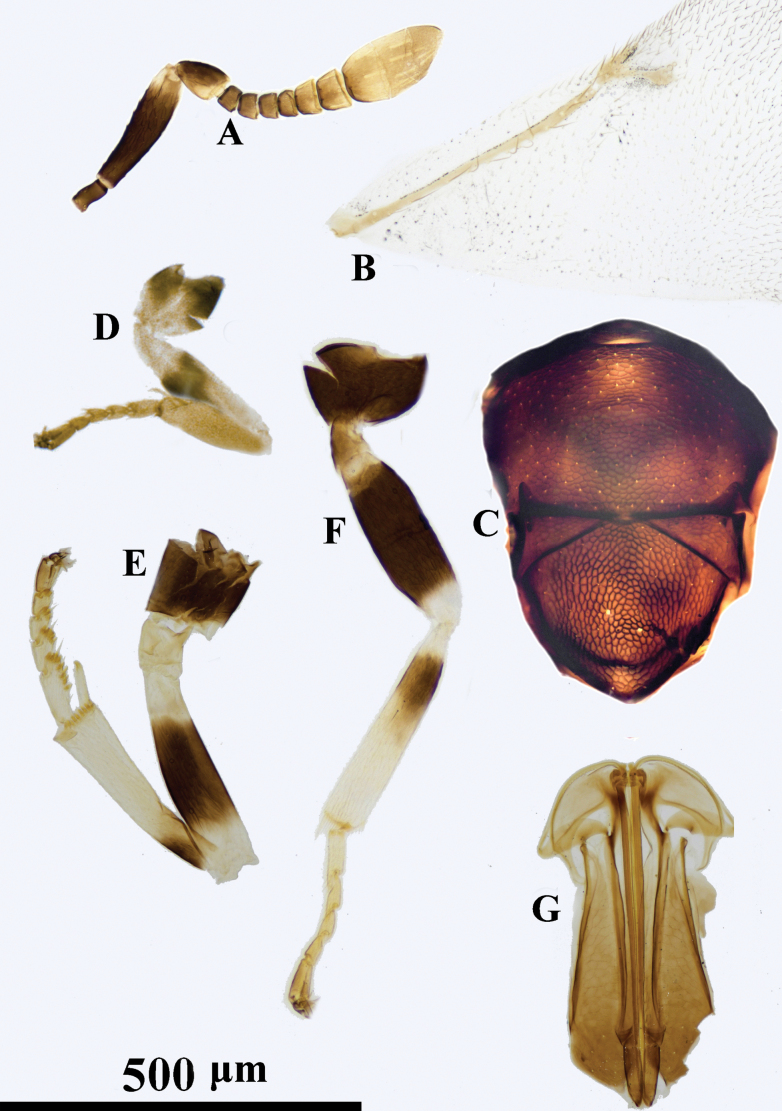
*Psyllaephagusclavus* sp. nov. ♀ **A** antenna **B** fore wing **C** mesonotum **D** fore leg **E** mid leg **F** hind leg **G** ovipositor.

Female. Body length ~ 1.0 mm; all coxae dark brown, but apical ~ 1/3 of fore coxa yellow; femora marked with dark brown (Fig. 3D–F); all funicular segments transverse; clava longer than F3–F6 combined (Fig. 3A); sculpture on scutellum deeper than that on mesoscutum (Fig. 3C); ovipositor not exserted (Fig. 3G), OL ~ 1.5× MT.

#### Description.

**Female. *Body*** length ~ 1.0 mm, with metallic green-blue sheen; basal 1/3 of tegula yellow, remainder dark brown; scape and pedicel except apices dark brown, F1–F4 yellowish brown, F5, F6, and clava brownish yellow; all coxae dark brown except apex of fore coxa yellow; basal ~ 1/2 of fore femur (Fig. 3D), mid femur except apices and hind femur medially dark brown; all tibiae yellow except mid and hind tibiae basally each with a dark brown spot; all tarsi yellow except apices dark brown; wings hyaline, venation brown.

***Head*** with reticulate sculpture, HW ~ 3× FV width, and ~ 2.9× HL; ocelli forming obtuse angle of slightly more than 90°; OCL ~ 1.3× diameter of posterior ocellus; OOL ~ 0.2× diameter of posterior ocellus; POL ~ 3.9× diameter of posterior ocellus; antenna with scape 3.7× as long as wide; pedicel ~ 1.5× as long as wide; F1–F6 broader than long; clava 3-segmented, ~ 2.0× as long as wide, nearly as long as F2–F6 combined. Relative measurements: HW 16; HL 5.3; FV 7; OCL 1.7; OOL 0.3; POL 6.2; SL 6.9; SW 1.9.

***Mesosoma*.** Scutellum slightly convex; mesoscutum dorsally with reticulate sculpture similar to that on frontovertex; sculpture on scutellum similar but clearly deeper than that on mesoscutum; marginal vein ~ 2.0× as long as wide; postmarginal vein present, ~ 2.6× as long as wide; stigmal vein 4.0× as long as wide, STV ~ 2.7× PMV (Fig. 3B); mid tibia spur 0.5× as long as basitarsus. Relative measurements: FWL 33; FWW 19.5.

***Metasoma*** obviously shorter than mesosoma; cercal plates located on the apical 2/5 of metasoma, hypopygium reaching ~ 2/3 of metasoma; ovipositor slightly exserted or hidden (Fig. 3G); OL ~ 5.6× GL, and 1.9× MT.

**Male.** Unknown.

#### Etymology.

The specific name refers to its enlarged clava.

#### Host.

Unknown.

#### Distribution.

China (Xinjiang).

#### Comments.

*Psyllaephagusclavus* was erroneously treated as *P.nartshukae* Trjapitzin, 1986 in Tang et al. (2016). A detailed study of the morphological characters shows that it is an undescribed species. Using the keys in Trjapitzin (1989), it runs to *P.nartshukae* Trjapitzin but can be separated from *P.nartshukae* by F1–F6 broader than long (in *P.nartshukae*, F1–F5 longer than broad); clava obviously longer than F3–F6 combined(in *P.nartshukae*, clava slightly shorter than F4–F6 combined, or as long as F4–F6 combined).

### ﻿ Psyllaephagus colposceniae

Taxon classificationAnimaliaHymenopteraEncyrtidae

Trjapitzin, 1969

4410CD57-4CA4-5D55-BCF1-EA3FCD55A656

Fig. 4

Psyllaephagus colposceniae Trjapitzin, 1969: 52. Holotype ♀, Moldova (ZISP), not examined.
Psyllaephagus colposceniae Trjapitzin, 1978: 236–328; 1989: 256; Myartseva 1979: 27–33; Tang et al. 2016: 63–78.

#### Material examined.

China – **Xinjiang** • 1♀, Karamay, 23.July.2012, by sweeping, leg. HY Hu group; 1♀, Karamay, 28.July.2014, by sweeping, leg. HY Hu group; 1♀, Hami, 1.Aug.2016, by sweeping, leg. HY Hu group; 4♀♀, 2♂♂, Altai, 11.Jun.2023, ex. *Colposcenia* sp. on *Tamarixramosissima*, leg. BY Zou.

#### Diagnosis.

**Figure 4.**
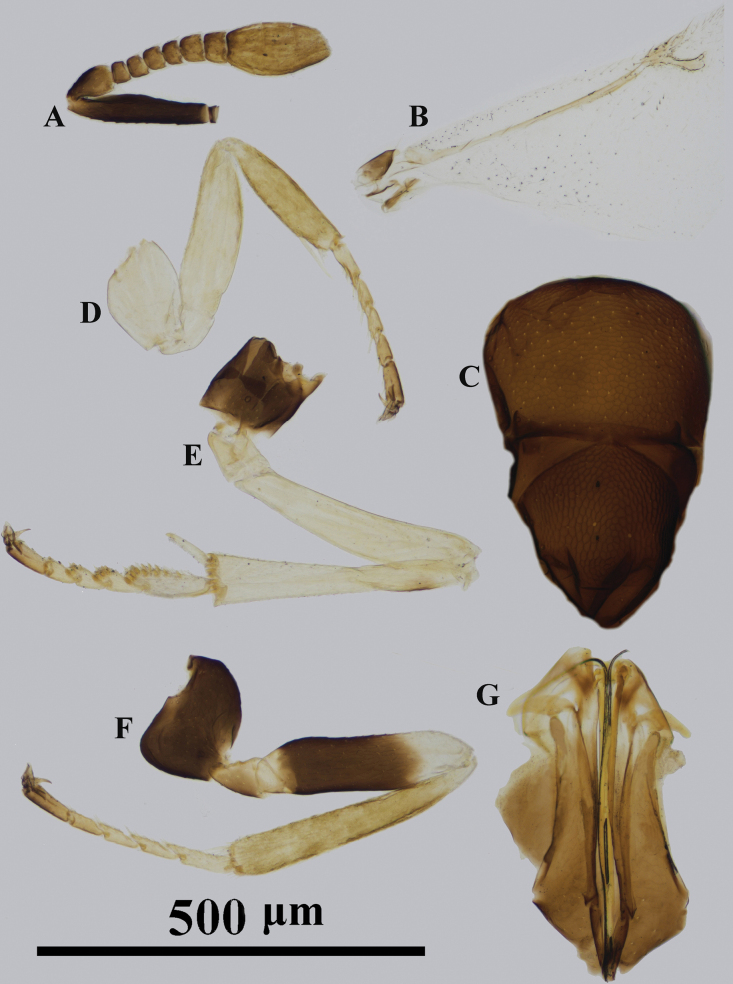
*Psyllaephaguscolposceniae* ♀ **A** antenna **B** fore wing **C** mesonotum **D** fore leg **E** mid leg **F** hind leg **G** ovipositor.

Female. Body length ~ 1.0 mm; mid and hind coxae dark brown, mid tibia with a dark brown spot at base, basal 3/4 of hind femur dark brown (Fig. 4E, F); ocelli forming angle of slightly less than 90°; all funicular segments transverse (Fig. 4A); mesoscutum and scutellum with contrasting sculpture, sculpture on scutellum somewhat deeper than that on mesoscutum; ovipositor hidden or slightly exserted, OL ~ 1.2× MT (Fig. 4G).

#### Description.

See Tang et al. (2016).

#### Host.

*Colposceniakiritshenkoi* Loginova, 1960 (Hemiptera: Aphalaridae).

#### Distribution.

China (Xinjiang); other countries: Kazakhstan, Moldova, Turkmenistan.

#### Comments.

The specimens studied here agree well with the original description of this species, except the slight variation in dimension of F2. In some specimens here, F2 is slightly shorter than broad (see also Myartseva 1979). We regard this difference falls within variation of a species.

### ﻿ Psyllaephagus densiciliatus

Taxon classificationAnimaliaHymenopteraEncyrtidae

Tan & Zhao, 1999

54830D80-6EC7-53A7-B61F-3852E299F106

Fig. 5

Psyllaephagus densiciliatus Tan & Zhao, 1999: 174–175. Holotype ♀, China, Yunnan (CNEP), photos of holotype examined.

#### Material examined.

China – **Hainan** • 2♀, Nada, 1.May.1964, leg. DX Liao; 3♀♀, 1♂, Wuzhishan, 21.Jun.1999, leg. CD Zhu; **Yunnan** • 2♀♀, Mengla, 23.July.2004, leg. YZ Zhang; **Guangxi** • 3♀♀, Fangcheng, 18.July.2009, leg. YZ Zhang.

#### Diagnosis.

**Figure 5.**
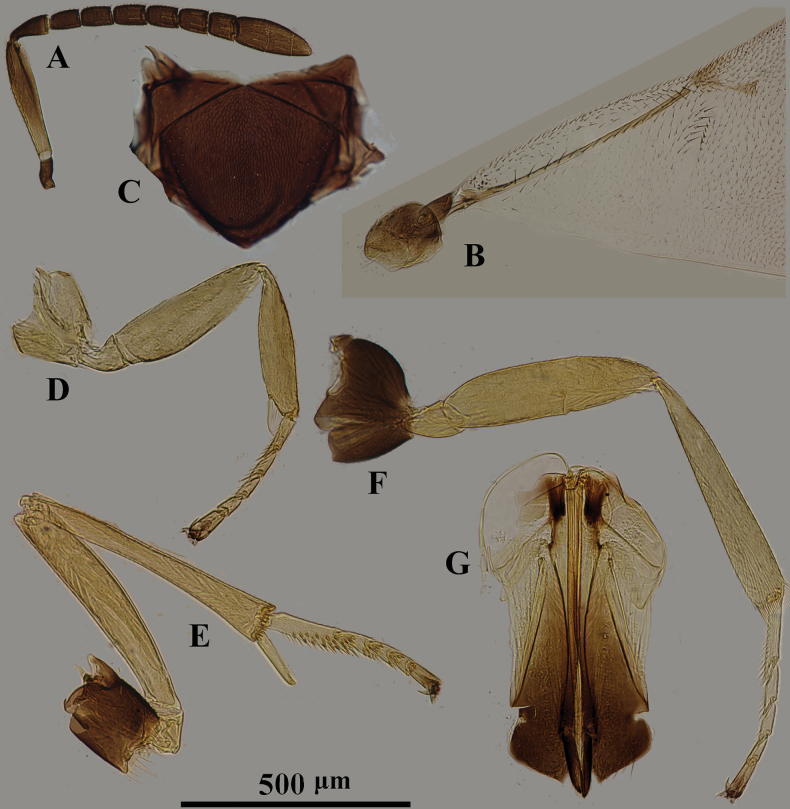
*Psyllaephagusdensiciliatus* ♀ **A** antenna **B** fore wing **C** scutellum **D** fore leg **E** mid leg **F** hind leg **G** ovipositor.

Female. Body length ~ 2.2 mm; legs yellow, but mid and hind coxae dark brown (Fig. 5E, F); antenna with all funicular segments longer than broad, F6 sometimes subquadrate (Fig. 5A); ovipositor slightly exserted; OL ~ 1.2× MT.

#### Description.

**Female. *Body*** length ~ 2.2 mm, metallic green; frontovertex and face with green-violet sheen. Antenna scape and pedicel dark brown, funicular segments brown but F6 yellow; mesoscutum with a coppery sheen; axilla and scutellum with blue sheen; basal 1/2 of tegula yellow, apex dark brown; mesopleuron metallic green mixed with golden sheen; legs yellow, but mid and hind coxae dark brown; basal 1/4 of metasoma with a metallic green sheen, remainder coppery; wings hyaline, venation brown.

***Head*** ~ 3.0× as wide as frontovertex; head with reticulate sculpture; ocelli forming angle of ~ 90°; OCL ~ 1.7× diameter of posterior ocellus; OOL ~0.6× diameter of posterior ocellus; POL ~ 3.8× diameter of posterior ocellus; mandible with one tooth and a broad truncation; antenna with scape ~ 4.9× as long as wide; pedicel ~ 2.0× as long as wide; all funicular segments longer than wide; clava 3-segmented, 2.5× as long as wide, slightly shorter than F4–F6 combined. Relative measurements: HW 32; HL 14; FV 9.7; OOL 0.8; POL 7.2; OCL 2.4; SW 3.1; SL 14.

***Mesosoma*.** Dorsum distinctly convex; MSW ~ 1.5× MSL; scutellum ~ 1.1× as long as wide; mesoscutum dorsally with reticulate sculpture similar to that on frontovertex; sculpture on scutellum similar to that on mesoscutum but clearly deeper; fore wings ~ 2.2× as long as wide; marginal vein quadrate; postmarginal vein short, ~ 1.3× as long as wide; stigmal vein 4.9–as long as wide, STV ~ 3.3× PMV; mid tibial spur 0.7× as long as basitarsus. Relative measurements: FWL 74; FWW 33.

***Metasoma*** slightly longer than mesosoma; cercal plates located in posterior 3/5 of metasoma; hypopygium reaching ~3/4 of metasoma; ovipositor slightly exserted; OL ~ 6.1× GL, and 1.2× MT.

**Male.** Body length ~ 1.1 mm, generally similar to female, but mesoscutum with coppery sheen; ocelli forming an obtuse-angled triangle; clava unsegmented.

#### Variation.

A little variation was found in coloration of the scape: the scape is not always totally dark brown, sometimes the base is yellow (Fig. 5A).

#### Host.

Unknown.

#### Distribution.

China (Guangxi, Hainan, Yunnan).

### ﻿ Psyllaephagus elaeagni

Taxon classificationAnimaliaHymenopteraEncyrtidae

Trjapitzin, 1967

414C0552-1FF0-5821-8F12-9238CABD9887

Fig. 6

Psyllaephagus elaeagni Trjapitzin, 1967: 192. Holotype ♀, Armenia (ZISP), not examined.
Psyllaephagus bachardenicus Myartseva, 1980: 50. Holotype ♀, Turkmenistan (ZISP). Synonymized by Japoshvili and Noyes (2005: 141).
Psyllaephagus rubriscutellatus Myartseva, 1981: 14. Holotype ♀, Turkmenistan (ZISP). Synonymized by Japoshvili and Noyes (2005: 141).
Psyllaephagus elaeagni : Tang et al. 2016: 65.

#### Material examined.

China – **Xinjiang** • 7♀♀, Qitai, 29.July.2012, leg. HY HU’s group; 2♀♀, Hoboksar, 26.July.2014, leg. HY HU’s group; 11♀♀, Mori, 29.July.2016, leg. HY HU’s group.

#### Diagnosis.

**Figure 6.**
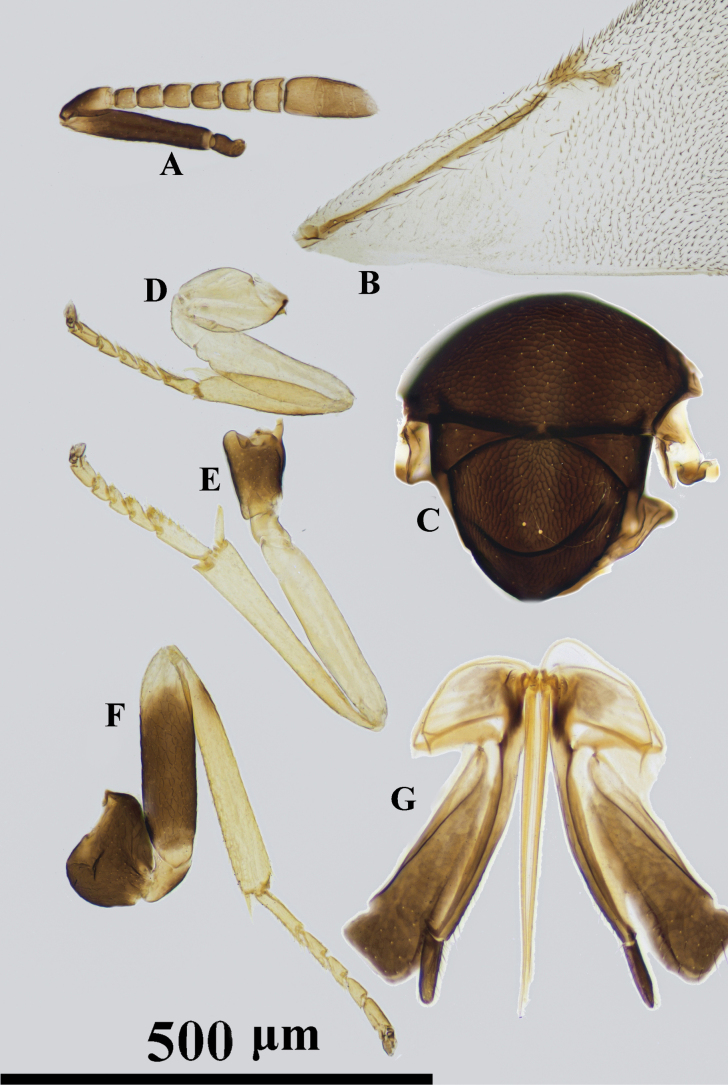
*Psyllaephaguselaeagni* ♀ **A** antenna **B** fore wing **C** mesonotum **D** fore leg **E** mid leg **F** hind leg **G** ovipositor.

Female. Mid and hind coxae dark brown, hind femur dark brown, basal of hind tibia with dark brown (Fig. 6F); F2–F5 longer than wide, F1 and F6 quadrate (Fig. 6A); mesoscutum and scutellum with contrasting sculpture, somewhat deeper than that on mesoscutum (Fig. 6C); metasoma approximately as long as mesosoma, OL ~ 1.3× MT.

#### Description.

See Tang et al. (2016).

#### Host.

Unknown.

#### Distribution.

China (Xinjiang); other countries: Armenia, Turkmenistan, Kazakhstan.

### ﻿ Psyllaeohagus guangxinesis

Taxon classificationAnimaliaHymenopteraEncyrtidae

Zu, 2021

E1DE3144-2724-5F43-9D5B-846ECCA44DC5

Fig. 7

Psyllaeohagus guangxinesis Zu, in Wu et al. 2021: 1–10. Holotype ♀, China, Guangxi (TJAU), examined by BYZ.

#### Material examined.

China – **Guangxi** • Holotype ♀, Guilin, 29.Aug.2018, ex. *Macrohomotomasinica* on *Ficusconcinna*, leg. GH Zu & Y Chen (TJAU); **Yunnan** • 35♀♀, 9♂♂, Mengla, 16.May.1973, ex. *Macrohomotomagladiatean* on *Ficus* sp., leg. DX Liao; **Fujian** • 5♀♀, 3♂♂, Huian, 16.Apr.2017, ex. *Macrohomotomagladiatean* on *Ficus* sp., leg. YG Qin & M Xiong; **Jiangxi** • 21♀♀, 17♂♂, Ganzhou, 20.Jul.2021, ex. *Macrohomotomagladiatean* on *Ficus* sp., leg. YZ Zhang; **Sichuan** • 5♀♀, 3♂♂, Chengdu, 21.Jun.2018, ex. *Macrohomotomagladiatean* on *Ficus* sp., leg. HL Li.

#### Diagnosis.

**Figure 7.**
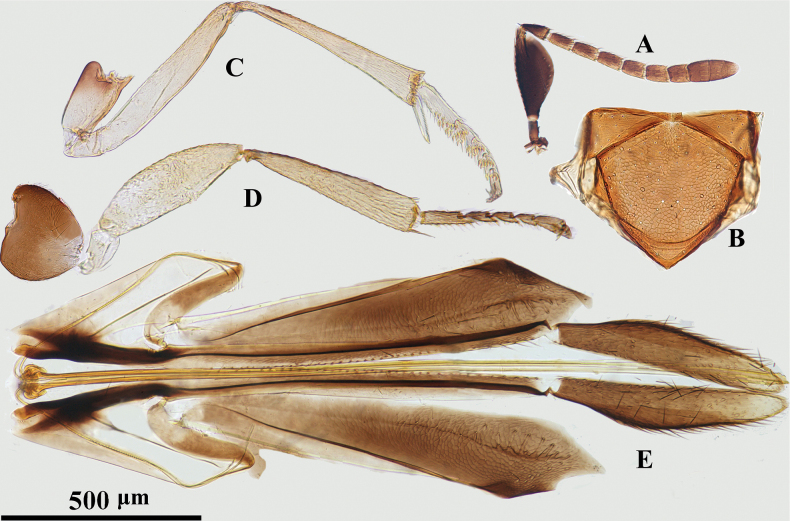
*Psyllaeohagusguangxinesis* ♀ **A** antenna **B** scutellum **C** mid leg **D** hind leg **E** ovipositor.

Female. Body length ~ 2.3 mm; legs yellow, but sometimes basal 1/3 of all hind femora dark brown (Fig. 7D); scape strongly flattened and expanded, less than 2.5× longer than broad (Fig. 7A); metasoma ~ 1.3× longer than mesosoma; ovipositor exserted, exserted part ~ 1/2 of metasoma; OL ~ 3.8× MT.

#### Variation.

A little variation was found in coloration of the mid and hind coxae: the mid coxa is not always completely yellow, sometimes the base can be dark brown (Fig. 7C); the hind coxa varies from partly to totally dark brown (Fig. 7D).

#### Description.

See Wu et al. (2021).

#### Hosts.

*Macrohomotomasinica* Yang & Li, 1984 (Hemiptera: Homotomidae).

#### Distribution.

China (Guangxi, Guangdong, Jiangxi, Yunnan, Fujian).

### ﻿ Psyllaephagus latiscapus

Taxon classificationAnimaliaHymenopteraEncyrtidae

Xu, 2000

D704EE1C-C096-5FC7-A6A9-E53AC4C5E771

Figs 8–10 , 11

Psyllaephagus latiscapus Xu, in Xu et al. 2000a: 39. Holotype ♀, China, Zhejiang (ZUIE), examined by YZZ.
Psyllaephagus latiscapus Xu: Tang et al. 2016: 69; Wu et al. 2021: 7.

#### Material examined.

China – **Zhejiang** • ***Holotype*** ♀, Hangzhou, 26.V.1957, ex. *Pachypsyllaceltidisgemma*, 5720-9, lge. QH Chen (ZUIE); **Shandong** • 4♀♀, 1♂, Qingdao, 24 Oct. 1962, ex. *Pachypsyllaceltidisgemma* on *Celtissinensis*, leg. QH Chen.

#### Diagnosis.

**Figures 8–10.**
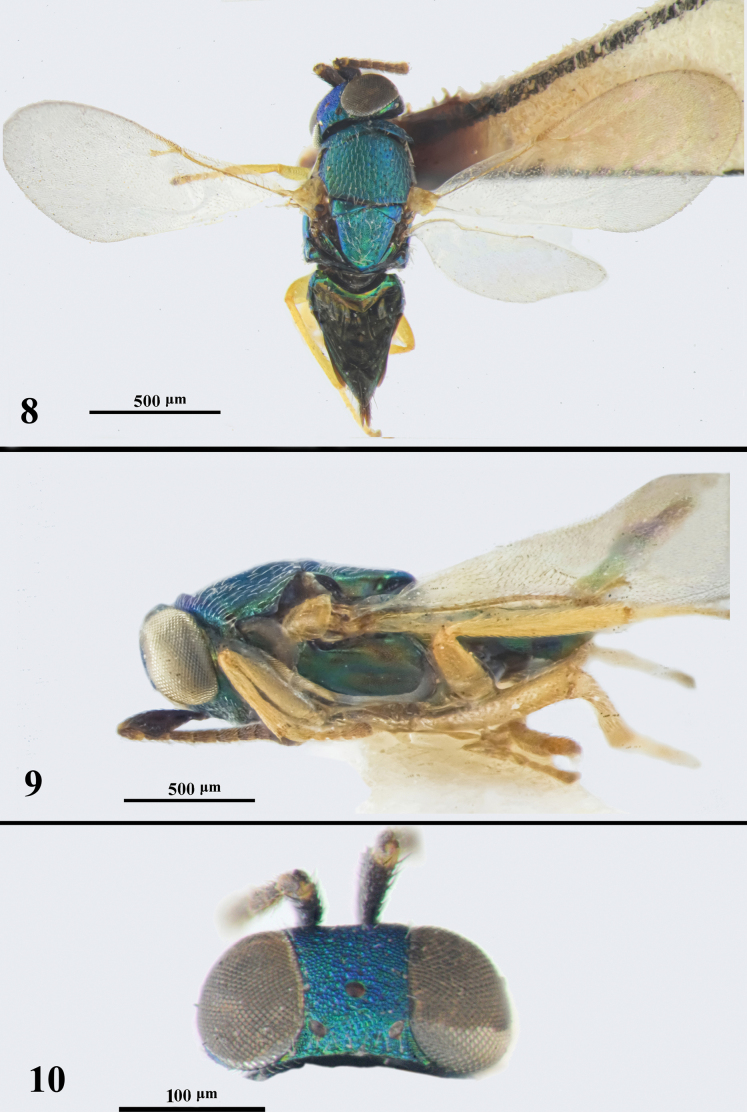
*Psyllaephaguslatiscapus* ♀ **8** body in dorsal view **9** body in lateral view **10** head in dorsal view.

**Figure 11.**
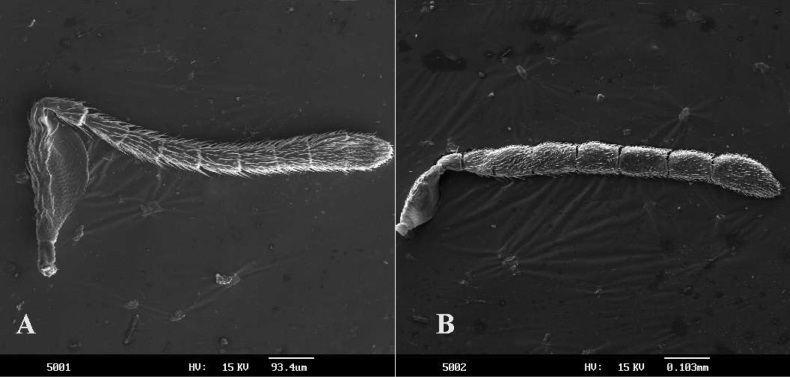
*Psyllaephaguslatiscapus***A** antenna ♀ **B** antenna ♂.

Female. Body length ~ 2.0 mm, head with metallic blue; tegulae entirely pale yellow; antenna with scape strongly expanded, ~ 2.3× as long as wide (Fig. 11A); postmarginal vein nearly as long as stigmal vein (Fig. 8); mid and hind coxae dark brown; ovipositor exserted, the exserted part ~ 1/5 of metasoma. OL ~ 1.5× MT.

#### Description.

See Xu et al. (2000a).

#### Host.

*Pachypsyllaceltidisgemma* Riley, 1885 (Hemiptera: Aphalaridae).

#### Distribution.

China (Zhejiang, Shandong, Fujian, Yunnan).

#### Comments.

The material above was treated as *P.punctatus* by Zhang (2001) in his thesis, which was not formally published but later cited by Tang et al. (2016) and Wu et al. (2021).

### ﻿ Psyllaephagus longifuniculus

Taxon classificationAnimaliaHymenopteraEncyrtidae

Xu, 2000

8983B009-24CC-5366-90CD-D06099B029CE

Fig. 12

Psyllaephagus longifuniculus Xu, in Xu et al. 2000: 40. Holotype ♀, Shaanxi (ZUIE), examined by YZZ.

#### Material examined.

China – **Shanxi** • ***Holotype*** ♀, Wugong, 21.Aug.1954, Dept. Plant Protection, Northwest Agric. Univ., ex. *Thysanogynalimbata*, C5526-1a (ZUIE); China – **Zhejiang** • 6♀♀, Lishui, 16.Jun.1981, leg. DX Liao; **Beijing** • 8♀♀, 5♂♂, 11.Nov.1995, ex. *Thysanogynalimbata*, leg. YZ Zhang; 9♀♀, 13♂♂, 09.Jun.2017, ex. *Thysanogynalimbata*, leg. YZ Zhang.

#### Diagnosis.

**Figure 12.**
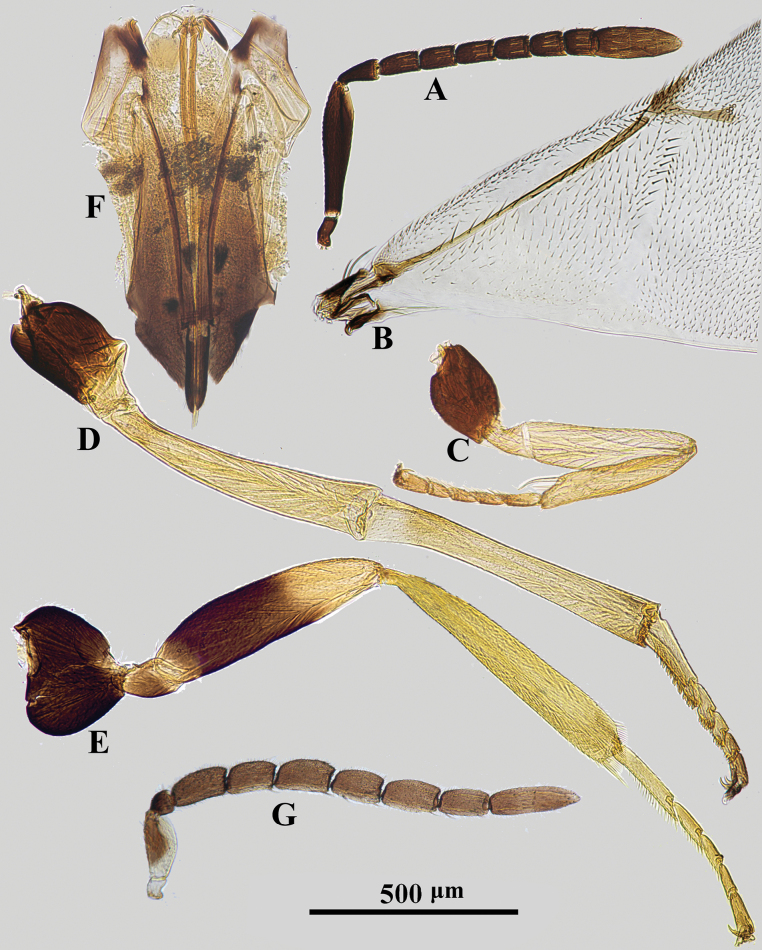
*Psyllaephaguslongifuniculus***A** antenna ♀ **B** fore wing ♀ **C** fore leg ♀ **D** mid leg ♀ **E** hind leg ♀ **F** ovipositor ♀ **G** antenna ♂.

Female. All coxae dark brown; F1 at least as long as or slightly longer than pedicel; F1–F5 obviously longer than broad, F6 slightly longer than broad or subquadrate (Fig. 12A); OL ~ 1.5× MT.

#### Description.

**Female. *Body*** length ~ 2.2 mm, with blue green sheen; basal 1/2 of tegula pale yellow, the rest dark brown; antenna completely dark brown; all coxae dark brown; hind femur dark brown, but apical ~ 2/5 yellow; wings hyaline, venation brown (Fig. 12B).

***Head*** with reticulate sculpture, HW ~ 3.0× HL in dorsal view, 2.9× as wide as frontovertex; ocellus forming an obtuse triangle, angle slightly more than 90°; OCL ~ 0.8× diameter of posterior ocellus; OOL ~ 1.1× diameter of posterior ocellus; POL ~ 4.0× diameter of posterior ocellus; mandible with one tooth and a broad truncation; antenna with scape slightly expanded and ~ 4.8× as long as wide; pedicel ~ 1.8× as long as wide; F1–F5 longer than broad, F6 quadrate; clava 3-segmented, approximately as long as F4–F6 combined, nearly 3.0× as long as wide (Fig. 12A). Relative measurements: SL 14.4; SW 3.

***Mesoscutum*** slightly convex; mesoscutum and scutellum with reticulate sculpture similar to that on frontovertex; MSW ~1.5× MSL, and the center of the posterior margin protrudes backward to cover junction of axillae; marginal vein subquadrate, slightly longer wide, stigmal vein ~ 6.5× as long as wide, postmarginal vein ~ 2.7× as long as wide, PMV ~ 0.4× STV; mid tibia spur 0.9×–as long as basitarsus. Relative measurements: FWL 64; FWW 27.

***Metasoma*** slightly shorter than mesosoma; cercal plates located at the halfway point of the metasoma; hypopygium with apex reaching ~ 2/3 of metasoma; ovipositor not exserted; OL ~ 5.3× GL, and 1.2× MT.

**Male.** Body length ~ 1.6 mm; generally similar to female except color and structure of antenna, antenna pale brown, scape and pedicel short, funicular segments protrude laterally, clava unsegmented (Fig. 12G).

#### Variation.

In *P.longifuniculus*, a little variation was found in lengths of F1 and F6: F1 is not always shorter than the pedicel, sometimes it is ~ 1.1× as long as pedicel; F6 is sometimes quadrate (Fig. 12A).

#### Host.

*Thysanogynalimbata* Enderlein, 1926 (Hemiptera: Carsidaridae).

#### Distribution.

China (Beijing, Shaanxi, Zhejiang).

### ﻿ Psyllaephagus longiventris

Taxon classificationAnimaliaHymenopteraEncyrtidae

Trjapitzin, 1964

109F012A-D3DA-508B-B159-18BC71713830

Fig. 13

Psyllaephagus longiventris Trjapitzin, 1964: 237. Holotype ♀, Kazakhstan (ZISP), not examined.
Kaszabicyrtus acutigastris Szelényi, 1971: 390–391, Holotype ♀, Mongolia. Synonymized by Szelényi (1972: 372).
Psyllaephagus longiventris : Myartseva 1984: 143, 235–236; 1986: 216, 255, 257; Tang 2016: 70–72.

#### Material examined.

China – **Xinjiang** • 7♀♀, 2♂♂, Turpan, 03.Aug.2012, leg. HY Hu group; 11♀♀, 5♂♂, Xinjiang, Turpan, 11.Aug.2012, leg. HY Hu group. 5♀♀, Altai, 11.June.2023, leg. BY Zou.

#### Diagnosis.

**Figure 13.**
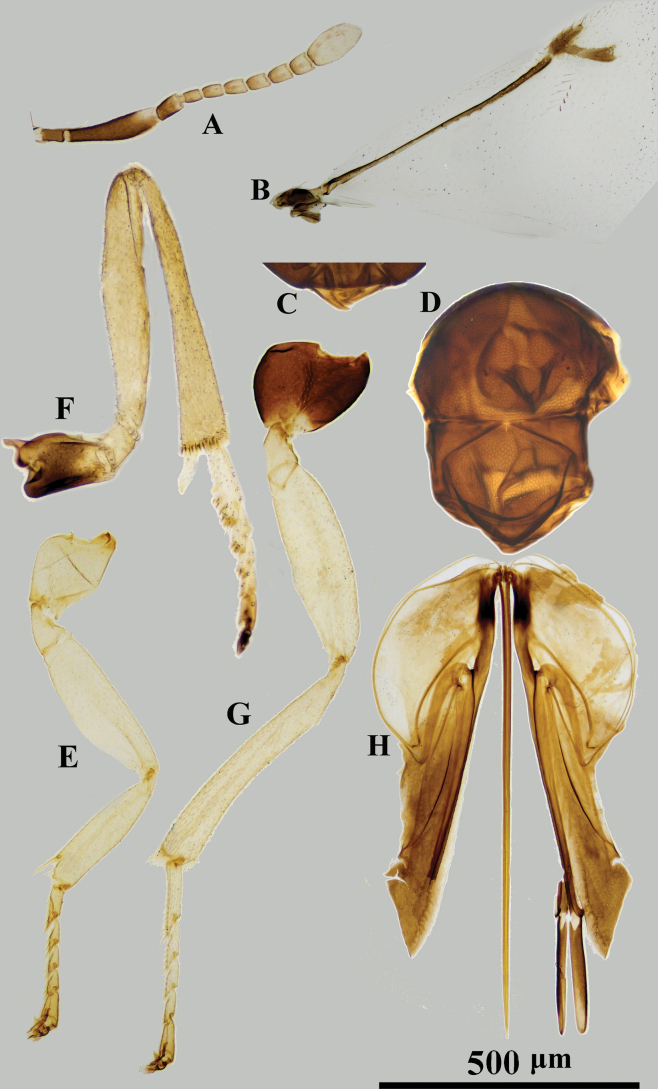
*Psyllaephaguslongiventris* ♀ **A** antenna **B** mandible **C** fore wing **D** mesonotum **E** fore leg **F** mid leg **G** hind leg **H** ovipositor.

Female. Legs yellow, but mid and hind coxae dark brown; ocelli forming right triangle; mandible with two teeth and a truncation (Fig. 13C); F1–F5 longer than broad (Fig. 13A); metasoma almost 2.0× as long as mesosoma; the ovipositor protrudes ~ 2/5 of metasoma (Fig. 13H); OL ~ 1.6× MT.

#### Description.

See Tang et al. (2016).

#### Host.

*Caillardiarobusta* Loginova, 1956 (Hemiptera: Aphalaridae).

#### Distribution.

China (Xinjiang); other countries: Kazakhstan, Mongolia, Turkmenistan, Uzbekistan.

### ﻿ Psyllaephagus obliquus

Taxon classificationAnimaliaHymenopteraEncyrtidae

Zou & Zhang sp. nov.

7852D023-C800-54E8-B9AF-72A05D8C4A1E

https://zoobank.org/F91EE53B-2AFB-4614-A7E8-19731147E1F6

Fig. 14

#### Type material.

***Holotype*** ♀ [on slide], China – **Guizhou**, Zunyi, 28.Aug.2017, ex *Edentatipsyllashanghaiensis*, leg. YZ Zhang and YG Qin (deposited in IZCAS). ***Paratypes*** 10♀♀, 2♂♂, same data as holotype.

#### Diagnosis.

**Figure 14.**
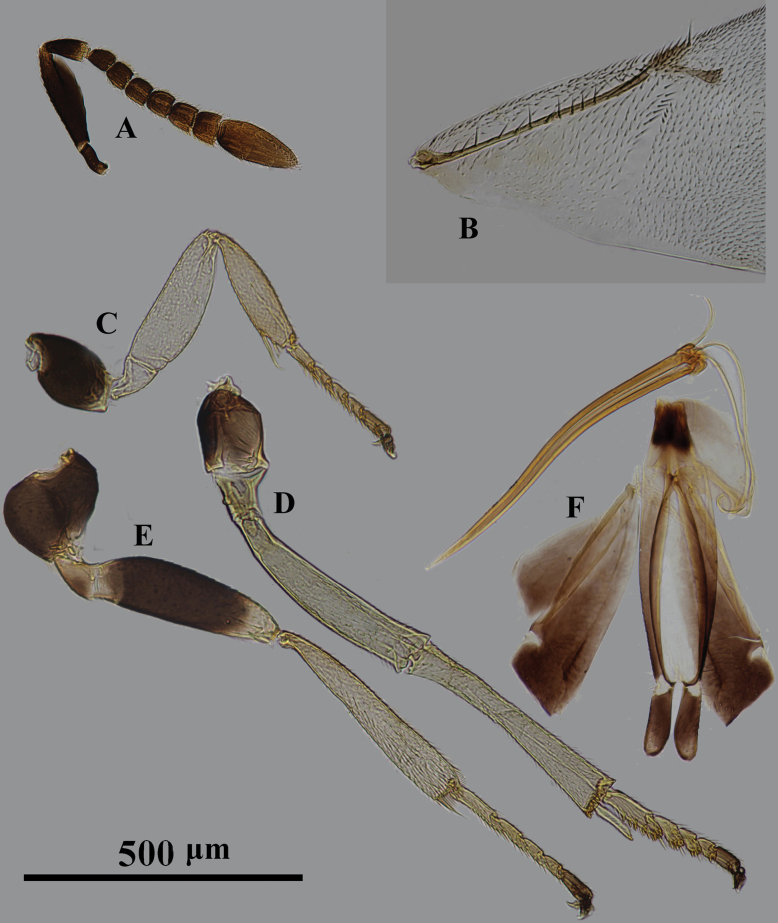
*Psyllaephagusobliquus* sp. nov. ♀ **A** antenna **B** fore wing **C** fore leg **D** mid leg **E** hind leg **F** ovipositor.

Female. Body length ~ 1.5 mm; all coxae dark brown and hind femora; scape distinctly expand, ~ 3.2× as long as wide; F6 transverse; clava strongly obliquely truncated at apex (Fig. 14A); OL ~ 1.4× MT.

#### Description.

**Female. *Body*** length ~ 1.8 mm, with blue-green sheen; basal ~ 1/2 of tegula yellow, remainder dark brown; scape nearly completely dark brown; pedicel dark brown except apex yellow; funiculus from dark brown to yellow brown; clava yellowish brown; all coxae, hind femur except apices dark brown; wings hyaline, venation brown (Fig. 14B).

***Head*** with reticulate sculpture, HW ~ 2.2× the HL in dorsal view, and ~ 2.8× wide as frontovertex; ocellus forming an angle ~ 100°; OCL ~ 1.2× diameter of posterior ocellus; OOL ~ 0.4× diameter of posterior ocellus; POL ~ 3.6× diameter of posterior ocellus; mandible with two teeth and a broad truncation; upper margin of torulus upper than lower margin of eye; scape distinctly expanded, ~ 3.2× as long as wide; pedicel ~ 2.7× as long as wide, approximately as long as F1 and F2 combined; F1 longer than broad, F2–F5 subquadrate, F6 almost wider than long; clava 3-segmented, nearly 2.2× as long as wide, approximately as long as F4–F6 combined; the apex of clava strongly obliquely truncated (Fig. 14A), the truncated parts ~ 1/2 clava length. Relative measurements: HW 21; HL 9; FV 7.5; OOL 0.8; POL 7.2; OCL 2.4; SW 2.6; SL 9.

***Mesoscutum*** slightly convex; mesoscutum and scutellum with reticulate sculpture similar to that on frontovertex; MSW ~ 1.4× MSL; scutellum and mesoscutum dorsally with reticulate sculpture similar to that on frontovertex; marginal vein ~ 1.3× as long as wide; stigmal vein ~ 4.1× as long as wide, postmarginal vein ~ 2.0× as long as wide, PMV ~ 0.3× of STV; mid tibia spur 0.8× as long as basitarsus. Relative measurements: FWL 50; FWW 22.

***Metasoma*** approximately as long as mesosoma; cercal plates located in the 1/2 of metasoma; hypopygium reaching ~ 3/4 of metasoma; ovipositor slightly exserted; OL ~ 5.3× GL, and 1.4× MT.

**Male.** Length 1.6–1.8 mm; generally similar to female in body coloration, except the antenna yellow; clava and flagellum have long bristles.

#### Host.

*Edentatipsyllashanghaiensis* Li & Chen, 2008 (Hemiptera: Psyllidae).

#### Distribution.

China (Guizhou, Shanghai).

#### Etymology.

The specific name refers to its strongly obliquely truncated clava.

#### Comments.

Using the key of Trjapitzin (1989), the species runs to *P.stenopsyllae* (Tachikawa, 1963), but can be separated from *P.stenopsyllae* by a combination of the following characters: scape ~ 3.2× as long as wide (in *P.stenopsyllae*, scape ~ 3.7× as long as wide); F2–F5 quadrate, F6 transverse (in *P.stenopsyllae*, F2–F5 obviously longer than wide, F6 quadrate); clava approximately as long as F4–F6 combined and the apex of clava strongly obliquely truncated (in *P.stenopsyllae*, clava slightly shorter than F4–F6 combined, and with apex more or less rounded). *Psyllaephagusobliquus* is similar to *P.nipponicus* (Ishii, 1928) but can be separated from *P.nipponicus* by pedicel ~ 2.5× as long as wide (in *P.nipponicus*, pedicel ~ 2× as long as wide); F1 ~ 1.2× as long as wide (in *P.nipponicus*, F1 quadrate); metasoma as long as mesosoma (in *P.nipponicus*, metasoma shorter than mesosoma).

### ﻿ Psyllaephagus ogazae

Taxon classificationAnimaliaHymenopteraEncyrtidae

Sugonjaev, 1968

447BF98A-0EC5-5BB2-81C0-EB44BD12236B

Fig. 15

Psyllaephagus ogazae Sugonjaev, 1968: 593. Holotype ♀, Uzbekistan (ZISP), not examined.
Psyllaephagus ogazae : Trjapitzin, 1989: 260; Tang et al. 2016: 76.

#### Material examined.

China – **Xinjiang** • 18♀♀, 7♂♂, Hoboksar, 29.Jul.2012, ex *Caillardianotata* on *Haloxylonammodendron*, leg. HY Hu’s group; 23♀♀, 9♂♂, Hoboksar, 06.Jun.2016, ex. *Caillardianotata* on *Haloxylon* sp., leg. HY Hu’s group; 11♀♀, Fukang, 03.Jun.2018, leg. HY Hu’s group.

#### Diagnosis.

**Figure 15.**
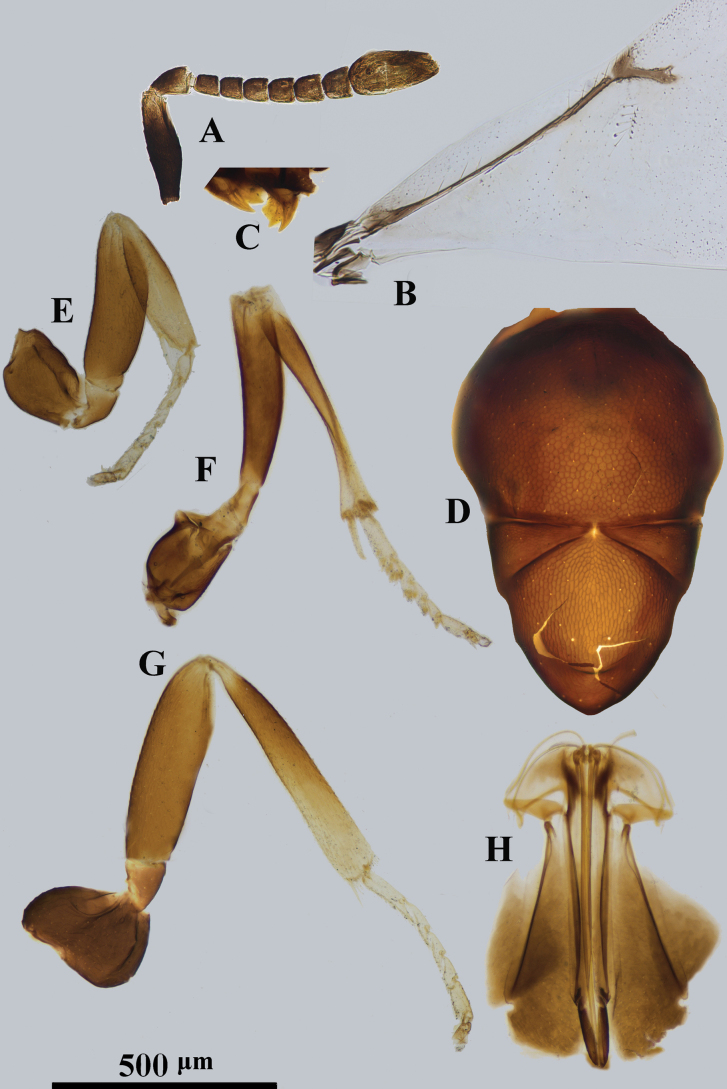
*Psyllaephagusogazae* ♀ **A** antenna **B** mandible **C** fore wing **D** mesonotum **E** fore leg **F** mid leg **G** hind leg **H** ovipositor.

Female. Body length 1.5–2.2 mm, with coppery green sheen; legs including all coxae, femora, and tibiae dark brown; ocelli forming a right-angled triangle; marginal vein and postmarginal vein almost absent (Fig. 15B); metasoma short, ~ 0.6–as long as mesosoma; OL ~ 6× GL, and 1.2× MT.

#### Description.

See Tang et al. (2016).

#### Variation.

Very little variation seen in our material: the hind tibia is not always dark brown, sometimes the apical 1/3 might be brownish yellow (Fig. 15G).

#### Host.

*Caillardiarobusta* Loginova, 1956 (Hemiptera: Aphalaridae).

#### Distribution.

China (Xinjiang); other countries: Kazakhstan, Mongolia, Tajikistan, Turkmenistan, Uzbekistan.

### ﻿ Psyllaephagus stenopsyllae

Taxon classificationAnimaliaHymenopteraEncyrtidae

(Tachikawa, 1963)

3BCA6677-5093-5BB1-9D42-DF06376EBA95

Fig. 16

Metaprionomitus stenopsyllae Tachikawa, 1963: 182. Holotype ♀, Japan, not examined.
Psyllaephagus stenopsyllae : Trjapitzin 1967: 177; Tan and Zhao 1999: 175; Xu et al. 2000: 9; Japoshvili et al. 2016: 392.

#### Material examined.

China – **Jiangxi** • 6♀♀, Ganzhou, 15.Oct.2012, ex. *Macrohomotomasinica*, leg. DY Huang; **Fujian** • 7♀♀, 2♂♂, Huian, 20.May.2012, ex. *Macrohomotomagladiatean* on *Ficus* sp., leg. YZ Zhang; **Sichuan** • 17♀♀, 3♂♂, Chengdu, 21.Jun.2018, ex. *Macrohomotomagladiatean* on *Ficus* sp., leg. YZ Zhang.

#### Diagnosis.

**Figure 16.**
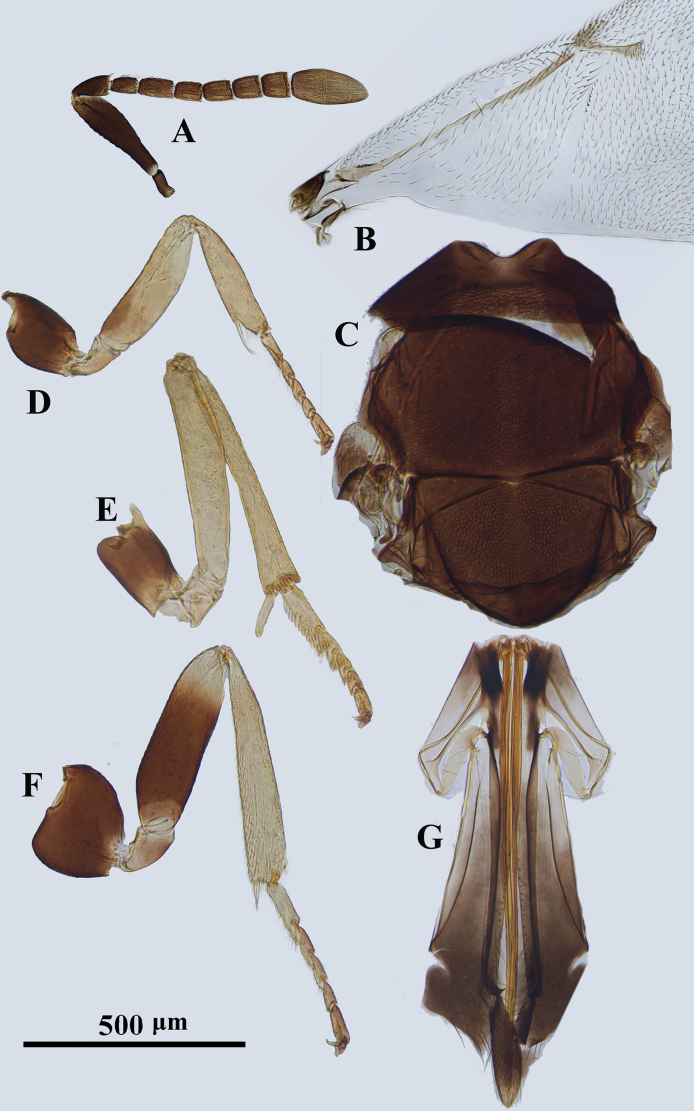
*Psyllaephagusstenopsyllae* ♀ **A** antenna **B** fore wing **C** mesonotum **D** fore leg **E** mid leg **F** hind leg **G** ovipositor.

Female. Body length ~ 2.0 mm; all coxae and hind femora dark brown (Fig. 16D–F); scape slightly expanded at apex; clava slightly short than F3–F6 combined (Fig. 16A); ovipositor exserted, exserted part ~ 1/5 metasomal length (Fig. 16G); OL ~ 1.9× as long as MT.

#### Description.

**Female. *Body*** length ~ 2.2 mm, head with metallic green sheen; mesoscutum and scutellum with bluish violet sheen; metasoma with coppery sheen; basal ~ 1/4 of tegula pale yellow, the remainder dark brown; antenna completely dark brown; all coxae dark brown; hind femur dark brown, but apical ~ 1/4 yellow; wings hyaline, venation brown (Fig. 16B).

***Head*** with reticulate sculpture, HW ~ 3.3× the HL in dorsal view, 3.1× wide as frontovertex; ocellus forms an obtuse triangle, angle ~ 110°; OCL ~ 1.0× diameter of posterior ocellus; OOL ~ 1.1× diameter of posterior ocellus; POL ~ 4.0× diameter of posterior ocellus; mandible with one tooth; scape slightly expanded and ~ 4.3× as long as wide; pedicel ~ 1.8× as long as wide; F1–F5 longer than broad, F6 quadrate; clava 3-segmented, nearly 2.3× as long as wide, shorter than F4—F6 combined. Relative measurements: SL 10.9; SW 2.5.

***Mesoscutum*** slightly convex; mesoscutum and scutellum with reticulate sculpture similar to that on frontovertex; MSW ~ 1.4× as MSL; marginal vein subquadrate, slightly longer wide, stigmal vein ~ 7.2× as long as wide, postmarginal vein ~ 1.6× as long as wide, PMV ~ 0.7× STV; mid tibia spur 0.8× as long as basitarsus. Relative measurements: FWL 51.6; FWW 36.3.

***Metasoma*** nearly as long as mesosoma; cercal plates located in middle of metasoma; hypopygium with apex reaching ~ 2/3 of metasoma; ovipositor exserted, exserted part of ovipositor ~ 0.1× as long as metasoma; OL ~ 4.8× GL, and 1.9× MT.

#### Hosts.

*Macrohomotomagladiatuum* Kuwayama, 1908, *Stenopsyllanigricornis* Kuwayama, 1910, *Triozasyzygii* Li & Yang, 1911 (Hemiptera: Triozidae).

#### Distribution.

China (Fujian, Hainan, Jiangxi, Taiwan); other countries: Japan, Iran.

### ﻿ Psyllaephagus tangae

Taxon classificationAnimaliaHymenopteraEncyrtidae

Zou & Zhang sp. nov.

326FD981-B794-58ED-9AD9-322BC9AA20EF

https://zoobank.org/04605f95-6f37-4b6f-be56-2f0fe70cb867

Fig. 17

Psyllaehagus nikolskajae Trjapitzin: Tang et al. 2016: 74 (misidentification).

#### Type material.

***Holotype*** ♀ [on slide], China – Xinjiang, Changji, 21.Jul.2012, by sweeping, leg. HY Hu’s group (deposited in ICXU); ***Paratypes*** 11♀♀, same data as holotype.

#### Diagnosis.

**Figure 17.**
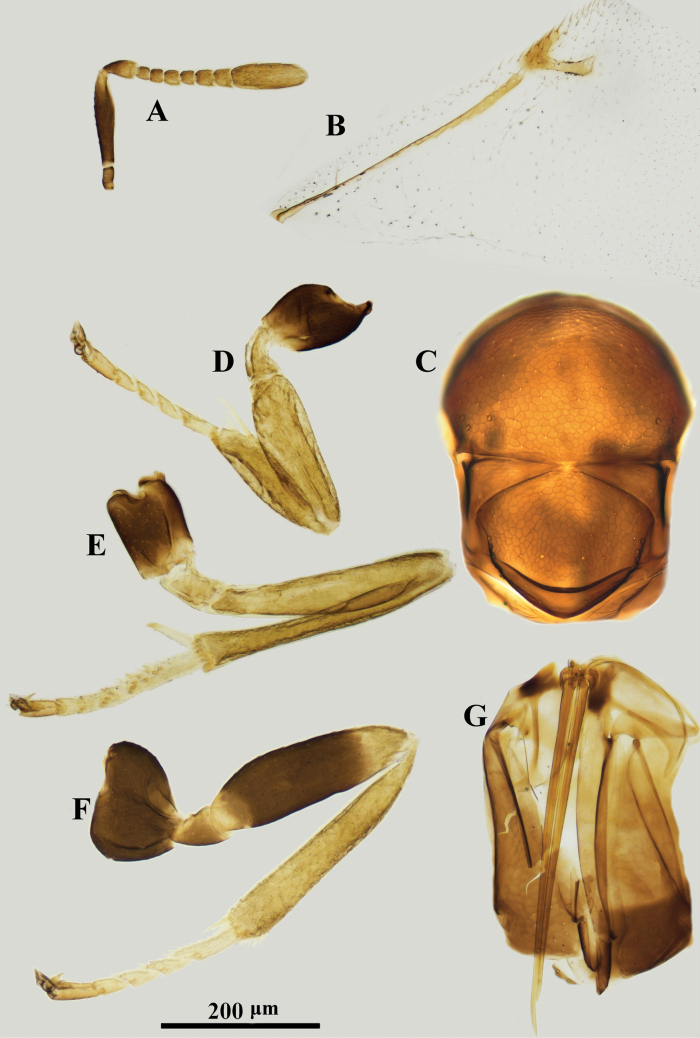
*Psyllaephagustangae* sp. nov. ♀ **A** antenna **B** fore wing **C** mesonotum **D** fore leg **E** mid leg **F** hind leg **G** ovipositor.

Female. Body length ~ 1.0 mm; all coxae dark brown, hind femur dark brown; F4 smaller than F3 in size (Fig. 17A); clava longer than F3–F5 combined; sculpture of scutellum with large cells in basal half and small cells near apex (Fig. 17C); OL ~ 1.2× MT.

#### Description.

**Female. *Body*** length ~ 1.0 mm, with greenish blue sheen; head and face generally dark with blue reflections; axillae with purple sheen; mesoscutum, axillae, and scutellum with bronze sheen; tegula entirely yellow; scape dark brown except apex yellow; basal 1/2 of pedicel dark brown, the remainder yellow; funiculus yellow; all coxae dark brown; hind femur dark brown but yellow at apex (Fig. 17D–F); wings hyaline, venation brown.

***Head*** with reticulate sculpture HW ~ 2.5× FV, HW ~ 3.2× HL, head finely reticulate, overlaid with regularly spaced shallow punctuations; ocelli forming an obtuse triangle, angle ~ 120°; OCL ~ 0.9× diameter of posterior ocellus; OOL ~ 0.6× diameter of posterior ocellus; mandible with two teeth and a broad truncation; antenna with scape 5.0× as long as wide; pedicel ~ 1.6× as long as wide; F1–F4 broader than long, F5 subquadrate, F6 quadrate; clava 3-segmented but not obvious; mandible with two teeth and a broad truncation. HW 17; HL 5.3; FV 7; OCL 1.4; OOL 0.96; SL 7.2; SW 1.4.

***Mesoscutum*** distinctly convex; dorsally with reticulate sculpture similar to that on frontovertex; sculpture of scutellum with large cells in basal half and small cells near apex; marginal vein punctiform, slightly longer than wide; postmarginal vein ~ 1.5× as long as wide; stigmal vein 4.7× as long as wide, STV ~ 3.0× PMV; mid tibia spur ~ 0.6× as long as basitarsus. FWL 31.6; FWW 18.1.

***Metasoma*** slightly shorter than mesosoma; cercal plates located in the posterior 1/2 of metasoma; hypopygium with apex reaching ~ 2/3 of metasoma; ovipositor not exserted (Fig. 17G); OL ~ 5.6× GL, and 1.2× MT.

**Male.** Unknown.

#### Etymology.

This species named after Miss Tang who contributed so much to the study of Encyrtidae.

#### Host.

Unknown.

#### Distribution.

China (Xinjiang).

#### Comments.

This species had been erroneously treated as *P.nikolskajae* in Tang et al. (2016). Further study of the morphological characters indicate it is an undescribed species. Using the key of Trjapitzin (1989), the species runs to *P.tokgaevi* Myartseva but differs as follows: clava nearly as long as F2–F6 combined (in *tokgaevi*, clava approximately as long as F3–F6 combined); OL ~ 5.6× GL (in *tokgaevi*, OL ~ 4.5× GL). *Psyllaephagustangae* is similar to *P.belanensis* but can be separated from *P.belanensis* by F4 smaller than F3 (in *P.belanensis*, F4 not smaller than F3 according to Trjapitzin 1968); F1 and F2 quadrate (in *P.belanensis*, F1 and F2 longer than wide); head as wide as thorax (in *P.belanensis*, head distinctly wider than thorax).

### ﻿ Psyllaephagus taiwanus

Taxon classificationAnimaliaHymenopteraEncyrtidae

Xu, 2000

0E8AF5CA-8E36-5729-BA80-B73A674A108B

Psyllaephagus taiwanus Xu, in Xu et al. 2000b: 9–10. Holotype ♀, China, Taiwan (TARI), not examined.

#### Diagnosis.

Female. Mid and hind coxae dark brown, hind femur and tibia dark brown, apical 1/3 of mid femora and basal 1/5 of mid tibia dark brown; clava as long as F3–F6 combined; sculpture of scutellum deeper and finer than that on mesoscutum; ovipositor slightly exserted.

#### Description.

See Xu et al. (2000b).

#### Host.

*Triozasyzygii* Li & Yang, 1911 (Hemiptera: Triozidae).

#### Distribution.

China (Taiwan).

### ﻿Key to Chinese species of *Psyllaephagus* (female)

**Table:** 

1	All coxae dark brown (Fig. 14C–E); rarely apex of fore coxa yellow (Fig. 3D)	**2**
–	At least fore coxa yellow (rarely basally dark brown)	**7**
2	All femora marked dark brown (Fig. 15E–G)	**3**
–	Only hind femur marked dark brown (Figs 12E, 16F, 17F)	**4**
3	Postmarginal vein nearly absent (Fig. 15C); F1–F5 slightly longer than broad; clava ~ as long as F4–F6 combined (Fig. 15A)	***P.ogazae* Sugonjaev**
–	Postmarginal vein present, ~ 1/3 of stigmal vein length; all funicular segments transverse, clava ca as long as F3–F6 combined (Fig. 3A)	***P.clavus* Zou & Zhang, sp. nov.**
4	Clava longer than F3–F6 combined; F4 smaller than F3 (Fig. 17A)	***P.tangae* Zou & Zhang, sp. nov.**
–	Clava shorter than F3–F6 combined; F4 not smaller than F3 in size	**5**
5	All funicular segments longer than wide; F1 as long as or slightly longer than pedicel; clava ~ 0.7× as long as F4–F6 combined (Fig. 12A)	***P.longifuniculus* Xu**
–	At least one funicular segment not longer than wide; F1 shorter than pedicel; clava not less than 0.8× F4–F6 combined	**6**
6	Ovipositor distinctly exserted, the exserted part of ovipositor ~ 1/5 of metasoma (Fig. 16G); F1–F5 longer than wide, F6 subquadrate; clava slightly shorter than F4–F6 combined, and with apex more or less rounded (Fig. 16A)	***P.stenopsyllae* Tchikawa**
–	Ovipositor not or slightly exserted, the exserted part of ovipositor less than 1/6 of metasoma; clava as long as F4–F6 combined, and with apex strongly obliquely truncated (Fig. 14A)	***P.obliquus* Zou & Zhang, sp. nov.**
7	Scape distinctly expanded, less than 2.5× as long as broad (Figs 7A, 11A)	**8**
–	Scape not or only slightly expanded, more than 3× as long as broad (Figs 6A, 13A)	**9**
8	Metasoma ~ 1.25× as long as mesosoma; ovipositor strongly exserted, exserted part of ovipositor ~ 1/3 of metasoma (Fig. 7E)	***P.guangxiensis* Zu**
–	Metasoma ~ as long as mesosoma, ovipositor slightly exserted, exserted part of ovipositor ~ 1/4 of metasoma (Figs 8, 9)	***P.latiscapus* Xu**
9	Metasoma nearly 2.0× as long as mesosoma; ovipositor obviously exserted, exserted part of ovipositor ~ 1/3 of metasoma (Fig. 13H)	***P.longiventris* Trjapitzin**
–	Metasoma slightly longer or shorter than mesosoma; ovipositor, if exserted, the exserted part of ovipositor not more than 1/5 of metasoma	**10**
10	F1–F5 transverse or at least broader than long (Fig. 4A)	**11**
–	F1–F5 longer than broad or quadrate (Fig. 2A)	**12**
11	Clava as long as F2–F6 combined; hind femur yellow at apex (Fig. 4F)	***P.colposceniae* Trjapitzin**
–	Clava as long as F3–F6 combined; hind femur dark brown, basal 1/3 of hind tibia dark brown	***P.taiwanus* Xu**
12	Ovipositor obviously exserted; the exserted part of ovipositor ~ 1/4 of metasoma; F6 quadrate (Fig. 2A); mid coxa yellow, but apical or so 1/3 dark brown (Fig. 2F)	***P.caillardiae* Sugonjaev**
–	Ovipositor slightly exserted, the exserted part of ovipositor no more than 1/5 of metasoma; F6 ~ 1.1× as long as wide; mid coxa entirely dark brown	**13**
13	All funicular segments longer than broad; hind femur yellow (Fig. 5F)	***P.densiciliatus* Tan & Zhao**
–	At least one segment of funicle not longer than broad; hind femur broadly dark brown (Figs 1G, 6F)	**14**
14	F1–F5 longer than broad, F6 quadrate (Fig. 1A); OL ~ 4.5× as long as GL (Fig. 1H)	***P.arenarius* Trjapitzin**
–	F1 and F5 quadrate, F2–F4 longer than broad, F6 broder than long (Fig. 6A); OL ~ 5.5× as long as GL (Fig. 6G)	***P.elaeagni* Trjapitzin**

## Supplementary Material

XML Treatment for Psyllaephagus

XML Treatment for Psyllaephagus arenarius

XML Treatment for Psyllaephagus caillardiae

XML Treatment for Psyllaephagus clavus

XML Treatment for Psyllaephagus colposceniae

XML Treatment for Psyllaephagus densiciliatus

XML Treatment for Psyllaephagus elaeagni

XML Treatment for Psyllaeohagus guangxinesis

XML Treatment for Psyllaephagus latiscapus

XML Treatment for Psyllaephagus longifuniculus

XML Treatment for Psyllaephagus longiventris

XML Treatment for Psyllaephagus obliquus

XML Treatment for Psyllaephagus ogazae

XML Treatment for Psyllaephagus stenopsyllae

XML Treatment for Psyllaephagus tangae

XML Treatment for Psyllaephagus taiwanus

## ﻿Additional information

### Author ORCIDs

Boyu Zou https://orcid.org/0009-0000-5022-8669

Hongying Hu https://orcid.org/0000-0002-2295-1072

Lanwei Zhang https://orcid.org/0000-0002-7829-8770

Yanzhou Zhang https://orcid.org/0000-0002-9713-5352

### Data availability

All of the data that support the findings of this study are available in the main text.
